# Amyotrophic Lateral Sclerosis and Novel Therapeutic Strategies

**DOI:** 10.1155/2012/798028

**Published:** 2012-12-19

**Authors:** Brett Morrison, Kenneth Hensley, Erik P. Pioro, Susanne Petri, Mahmoud Kiaei

**Affiliations:** ^1^Department of Neurology, Johns Hopkins University, Baltimore, MD 21205, USA; ^2^Department of Neurosciences, University of Toledo Medical Center, Toledo, OH 43614, USA; ^3^Department of Neurology, Neurological Institute, Cleveland Clinic, Cleveland, OH 44195, USA; ^4^Department of Neurology, Hannover Medical School, Hannover, 30625, Germany; ^5^Center for Translational Neuroscience, Department of Neurobiology and Developmental Sciences, College of Medicine, University of Arkansas for Medical Sciences, Little Rock, AR 72205, USA

Research and key discoveries in the field of amyotrophic lateral sclerosis (ALS) have exponentially increased since the announcement in 1993 of the first ALS-causing mutations in the gene for the well-studied antioxidant enzyme Cu,Zn superoxide dismutase (SOD1). The etiology of sporadic ALS largely remains unknown and the mechanisms of motor neuron degeneration are still being investigated. The only FDA drug approved for the treatment of ALS is riluzole with only modest benefit to patients, but multiple drugs are currently in the development pipeline and in human ALS clinical trials. In this special issue, K. Venkova-Hristova et al. thoroughly reviewed studies of experimental therapeutics in animal models of ALS, including specific examples of those that proceeded into human clinical trials. Since none of the ALS human clinical trials succeeded despite positive results in animal models, the question of “why” has been on everyone's mind, with efforts to develop superior alternatives. This review discusses the potential reasons for the universal failure of preclinical successes to translate into positive clinical outcomes. It has become obvious that the lack of understanding of the precise mechanisms of motor neuron degeneration presents a major obstacle in the development of effective therapy for ALS. This review discusses the details of several major pathogenic pathways in ALS and the efforts of various groups to block one toxic pathway at a time, ranging from oxidative stress to protein aggregation. The authors discuss the pros and cons of ALS models and propose simultaneous targeting of multiple pathways as a more efficient strategy, due to the multifactorial nature of ALS pathology. 

Several contributions in this special issue identify cellular physiological pathways that potentially exert neuroprotective effects if rationally manipulated for pharmacological mitigation against ALS. S. Petri et al., therefore, provide a review of a pivotal system called the Nrf2/ARE pathway which normally controls expression of “phase II genes,” a gene set relating to antioxidant and electrophilic detoxification, anti-inflammatory, and promitochondrial enzymes. Normal or induced Nrf2/ARE activity reduces neural vulnerability to oxidative stress, inflammatory factors, and mitochondrial dysfunction, but evidence suggests that the regulation of Nrf2/ARE is suboptimal in ALS. Hence, Nrf2 signaling pathway activation is a mechanism against at least three major and interrelated toxic pathways (oxidative stress, neuroinflammation, and mitochondrial dysfunction) in neurodegenerative diseases such as ALS, and it is an attractive and novel target. Accordingly, S. Petri and colleagues examine evidence that two blood-brain barrier penetrable CDDOs (2-cyano-3,12-dioxooleana-1,9-dien-28-oic acid), derivatives of the triterpenoid, an oleanic acid compound, function as remarkably potent Nrf2/ARE activators with proof of principal that slowed disease in ALS preclinical models. Further studies are required to investigate the mechanism and the pivotal role of Nrf2/ARE signaling pathway for neuroprotection in the spinal cord, specifically in motor neurons.

A review by E. Y. Achi and S. A. Rudnicki outlines the current state of knowledge about frontotemporal dysfunction and dementia (FTD) in ALS. Increasingly acknowledged in the current ALS literature, the incidence of FTD ranges from 7 to 22%, depending on the screening tests used. The authors describe the overlapping pathology and genetics seen in ALS and FTD, including the recently described C9orf72 mutations that appear to be a common cause of both familial ALS and FTD. They describe several clinical tests for FTD, while acknowledging that there is no current consensus on the best test to use. This review makes it clear that FTD is an important component of ALS that impacts the quality of life of patients and their caregivers. To date, there have been no clinical trials evaluating treatments of FTD in ALS patients, but based on FTD literature, the authors suggest selective serotonin reuptake inhibitors. It is unclear whether ALS patients with FTD will respond similarly to FTD patients in general, and therefore, there is little doubt that future clinical trials should be undertaken to evaluate the effectiveness of specific FTD treatments in ALS patients. In addition, the prevalence of FTD in ALS patients suggests that future clinical trials in ALS should incorporate measures of cognitive function, in addition to mortality, ALSFRS, and typical motor endpoints.

A major toxic pathway that has been extensively studied in ALS is neuroinflammation. Substantial evidence supports its detrimental effect on neurons in several neurodegenerative diseases. In this special issue, C. A. Lewis et al. discuss the neuroinflammatory responses in ALS and describe the cell and molecular components involved in neuroinflammation. The authors discuss the potential dual role of inflammation on motor neurons as both neuroprotective in the early stages of disease and neurotoxic later in disease and the challenges this presents when devising specific treatments for ALS. The authors specifically discuss a trophic effect of microglia that is induced by T cells, which could be a novel mechanism to alter disease progression by manipulating inflammatory pathways. 

The review by J. P. Crow et al. focuses on D-serine, a coagonist of the NMDA receptor which increases glutamate affinity via binding to the NR1 subunit and can, therefore, contribute to excitotoxic cell death, a major pathogenic mechanism in ALS. Based on findings of elevated D-serine level in the spinal cord of ALS transgenic mice and a delay in disease progression in ALS mice lacking the D-serine-producing enzyme serine racemase, it may represent an interesting therapeutic target. J. P. Crow et al. summarize current knowledge on D-serine regulation in vivo, in particular regarding the complex bidirectional function of serine racemase which can both produce and degrade serine. They review the literature on serine racemase knockout in in vivo models with specific focus on their previous and ongoing studies on the extent and function of serine expression in different tissues and cell types of transgenic ALS mice. Ultimately, the paper highlights the crucial importance of using appropriate methodology for reliable quantification of D-serine in tissue. Based on their own results as well as the current literature, they suggest an important role of D-serine in nonautonomous cell death of motor neurons mediated by glial cells. 

In the review by D. Krakora and colleagues, the authors examine the evidence for a “dying back” motor neuron injury in ALS. There have been several studies in the SOD1 transgenic mouse models of ALS suggesting that alterations in the muscles and motor axons occur before apparent pathology in motor neuron cell bodies. These pathologic studies are supported by several genetic and pharmacologic manipulations that were able to protect cell bodies while having no impact on the progression of weakness or mortality due to continued axon degeneration and muscle denervation. The authors also discuss studies in these models that found prolongation of lifespan following muscle-specific treatments. Taken together, these studies suggest a role for the peripheral axon, neuromuscular junction, and possibly muscle in the pathogenesis of ALS. The authors discuss several mechanisms that may be involved in this “dying back” motor neuron injury.

Further exploration of these mechanisms is important since effective treatments will need to address toxicity in both the soma as well as the distal axon. In their review, the authors describe several putative treatments, including delivery of growth factors to muscles, either directly or through mesenchymal stem cells, increasing energy supply to potentially hypermetabolic muscle, or exercise programs. Though several of these treatments have been shown to ameliorate the phenotype of SOD1 transgenic mice, they will likely need to be combined with treatments that protect motor neuron somata in order to produce sustained improvement in ALS patients. 

Perhaps the tightest bottleneck in translational ALS research now is selection of candidate therapeutics for clinical trials and conduct of the same. T. D. Levine et al. report a biomarker study of 27 ALS subjects taking the current “best practice” drug, riluzole. These subjects were randomized to receive pioglitazone (a PPAR agonist) and tretinoin (a retinoid), or placebo, for six months. Although the experimental treatment did not significantly slow functional decline on the standard ALSFRS-R scale, cerebrospinal tau concentrations decreased in the treatment arm compared to placebo. Interestingly, these researchers also found that phosphorylated neurofilament heavy chain (pNF-H) at baseline predicted faster rate of clinical decline. Taken together these findings suggest that strategies aimed at stabilizing the neuronal cytoskeleton might offer hope for ALS patients in the future and provide tantalizing clues about promising biomarkers to empower future clinical trials.

On a related biomarker topic, K. Kollewe et al. provide a thorough review of current magnetic resonance imaging (MRI) and functional MRI (fMRI) approaches to detect alterations of cortical networks in ALS during activated and task-free resting-state investigations. It is with the latter approach of fMRI-based resting state connectivity that the authors have contributed the original studies in ALS patients and identified disturbances not only in the Sensorimotor Network, as may be expected for a motor neuron degeneration, but also in the Default Mode Network, which interconnects brain regions involved in behavioral and cognitive functions. This is of relevance since ALS is now recognized to cause degeneration not only in motor but also in extramotor regions. They explain the advantages and utility of diffusion-tensor imaging (DTI), which is one of the most promising current MRI method to detect ALS-related changes in the white matter.

Altogether, papers in this special issue provide a series of comprehensive discussions and updates on the current status of research on pathogenic mechanisms in ALS and potential future therapeutics that may result from this research. Though our treatment options for ALS are currently very limited, advances in understanding the pathogenic mechanisms raise the hope for a more optimistic future for patients diagnosed with ALS. 

*Brett Morrison* *Brett Morrison* *Kenneth Hensley* *Kenneth Hensley* *Erik P. Pioro* *Erik P. Pioro* *Susanne Petri* *Susanne Petri* *Mahmoud Kiaei* *Mahmoud Kiaei*

